# Enhancing Mechanical Performance of High-Lignin-Filled Polypropylene via Reactive Extrusion

**DOI:** 10.3390/polym16040520

**Published:** 2024-02-14

**Authors:** Ruichen Wang, Xiangyu You, Shijie Qi, Ruiyun Tian, Huijie Zhang

**Affiliations:** College of Bioresources Chemical and Materials Engineering, Shaanxi University of Science of Science & Technology, Xi’an 710021, China; wang.ruichen@sust.edu.cn (R.W.); 210111015@sust.edu.cn (S.Q.); 230111094@sust.edu.cn (R.T.)

**Keywords:** lignin, polypropylene, mechanical performance, compatibility, high lignin content

## Abstract

Polypropylene (PP) is one of the most extensively used commodity plastics. In terms of eco-friendliness, it is worth considering preparing high-lignin-filled PP. This study explores the incorporation of high lignin content, derived from acetic acid lignin (AAL) and Kraft lignin (KL), into PP through twin-screw extrusion and injection molding. The challenge lies in maintaining mechanical performance. A compatibilizer—specifically, maleic anhydride-grafted polypropylene (MAPP)—is employed to enhance lignin–PP compatibility by chemically bonding with lignin and physically associating with the PP phase. Results indicate that KL maintains better dispersity than AAL. Compatibilizers with a high maleic anhydride (MA) level (≥0.8 wt.%) and moderate melt flow index (MFI) in the range of 60–100 g 10 min⁻¹ prove favorable in constructing a reinforced PP/KL network. Optimizing with 40 wt.% lignin content and 10 parts per hundred (pph) of compatibilizer yields blends with mechanical performance comparable to neat PP, exhibiting a notable increase in modulus and heat deflection temperature (HDT). Furthermore, utilizing PP/lignin blends can lead to a 20% reduction in expenses and approximately 40% reduction in PP-induced greenhouse gas (GHG) emissions. This approach not only reduces PP costs but also adds value to lignin utilization in a sustainable and cost-effective manner.

## 1. Introduction

Polypropylene (PP) stands out as the foremost commodity plastic globally, commanding an annual market value exceeding $100 billion. Its widespread use is attributed to remarkable mechanical strength, excellent chemical resistance, and facile processability, making it indispensable in diverse sectors such as packaging, household goods, medical devices, and automotive industries [1,2,3]. Despite its pivotal role, PP, derived entirely from non-degradable petroleum, raises environmental concerns due to its persistent nature. Addressing this challenge necessitates a crucial focus on PP recycling within its lifecycle. Unfortunately, PP’s recyclability is limited to approximately four cycles before it loses viability, ultimately ending up in landfills [4,5]. To mitigate the environmental impact and enhance sustainability, an alternative approach involves integrating PP with biomass as a feedstock. This strategy, particularly in the form of high-biomass-filled PP blends, presents a promising avenue. The incorporation of low-carbon-footprint biomass can significantly curtail reliance on fossil feedstocks and concurrently reduce associated greenhouse gas (GHG) emissions [6,7]. In this context, exploring the utilization of biomass-filled PP can be a viable and environmentally conscientious strategy for the plastic industry.

Lignin, the second-most abundant natural polymer after cellulose, is primarily obtained from the pulp and paper industry [8]. The annual production of such lignins, so-called technical lignins, is estimated to be over 50 million tons. However, a considerable portion of these lignins are traditionally incinerated for energy recovery [9,10,11]. Technical lignin, with a typical price range of USD 100–300 per metric ton, stands in contrast to the market value of polypropylene (PP), ranging from USD 1168–1515 per metric ton [12,13]. Thus, the incorporation of low-cost lignin into the PP matrix not only reduces the cost of final products but also mitigates environmental concerns associated with non-degradable PP, thereby unveiling the value-added potential of lignin. Previous studies have explored lignin/PP composites, but challenges persist in achieving high lignin content while maintaining mechanical properties. Ye et al. prepared the composites using hot pressing by mixing 0.5 wt.% of industrial pine Kraft lignin with PP, achieving mechanical performance comparable to neat PP [14]. Pregi et al. aimed for high-lignin-filled PP composites, employing extrusion and injection molding, resulting in a tensile strength of 20 MPa and Young’s modulus of 3 GPa at a high lignin content of 50% [15,16]. However, the higher lignin content adversely affected impact strength of the composites, leading to a significant decrease by 96%, which suggests a poor interaction between the PP phase and lignin molecules [16]. This emphasizes the importance of considering both an increase in lignin content and optimization of mechanical characteristics in the study of lignin/PP composites. Balancing these factors is crucial for achieving a composite material that combines the cost-effectiveness of lignin with the desired mechanical properties of PP, contributing to a more sustainable and economically viable solution.

Maleic anhydride-grafted PP (MAPP) is a common compatibilizer to improve the compatibility of different components in plastics. In previous reports, it has been established that both the concentration of lignin and addition of compatibilizing agent can greatly influence the properties of lignin/PP composites. To avoid significant deterioration in mechanical performance, it has been recommended that lignin content in the composite recipe should be kept below 10%, even when MAPP is utilized [17]. Abdelwahab et al. studied the possibility of using MAPP incorporated with ethylene butylacrylate glycidylmethacrylate (EBGMA) to improve the mechanical properties of polypropylene/lignin composites by enhancing the interfacial adhesion [18]. A higher lignin content of 30% was used, along with 10 phr of both compatibilizers. However, the impact strength still displayed a rapid decrease of 87% compared with neat PP. This implies that maintaining mechanical performance, especially for the impact strength, with high lignin-filled content in composites remains a significant challenge.

**Scheme 1:**
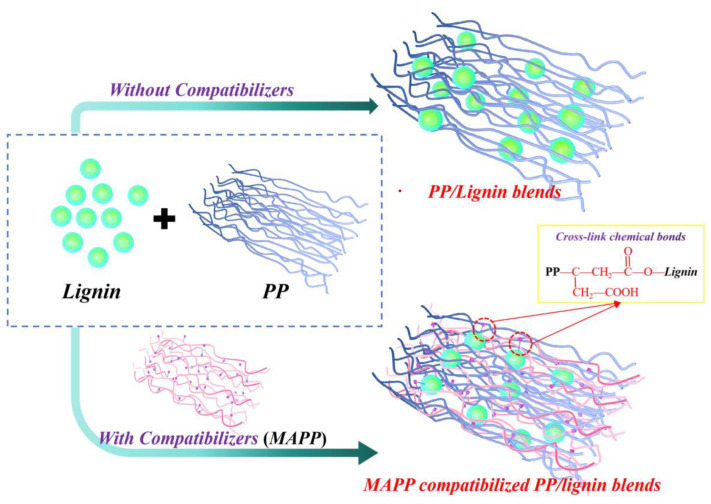
Scheme of PP/lignin and MAPP-compatibilized PP/lignin blends.

This study aims to maximize lignin content in the PP matrix without compromising mechanical properties. To achieve this goal, two types of lignins from different pulping processes were first selected and melt-blended with PP using twin-screw extrusion and injection molding techniques to prepare PP/lignin blends. Additionally, MAPP with varying maleic anhydride (MA) levels and melt flow index were introduced to enhance the compatibility of lignin and PP molecules (Scheme 1). The investigation encompassed mechanical performance assessments, morphological observations, thermodynamic measurements, rheological characterization, and evaluations of cost reduction and sustainability of the prepared lignin/PP blends.

## 2. Materials and Methods

### 2.1. Materials

The Tairipro K1035 Polypropylene homopolymer with a Melt Flow Index (MFI) of 25 g/10 min and a density of 0.90 g cm^−3^ was supplied by Formosa Chemicals (Taiwan, China). Technical Kraft lignin (KL) and acetic acid lignin (AAL) from Eucalyptus were kindly supplied by Guangzhou Yinnovator Biotech Co., Ltd., (Guangzhou, China). The lignins were used without purification, and the average particle sizes of KL and AAL were 11.1 μm and 32.4 μm, respectively (Figure S2). KL and AAL presented molecular weights of 6.3 and 5.9 kDa, with a hydroxyl group content of 4.3 and 0.2 mmol g^−1^, respectively (Table S1). Four different types of maleic anhydride-functionalized polypropylene—Exxelor^TM^ PO 1015 (CPA1), 1020 (CPA2) Polymer Resin, Coace^®^ B1 (CPA3), and B1R (CPA4)—were purchased from ExxonMobil Chemicals (Houston, TX, USA) and Coace Chemical Co., Ltd. (Xiamen, China). The substitution degree of maleic anhydride of these compatibilizers are listed in Table S2.

### 2.2. Preparation of PP/Lignin Blends

Powdery lignins and PP pellets were dried at 50 °C and 75 °C, respectively, for 48 h to remove moisture. The raw materials from various formulations were initially thoroughly mixed followed by an injection-molding process. This process utilized a lab-scale counter-rotating conical twin-screw extruder and injection molding machine (SJZS-10B and SZS-15, Ruiming Co., Ltd., Wuhan, China). The diameter of the screw used in the extruder decreased from 29 to 10 mm along the barrel length of 250 mm from feed to die. The processing conditions were as follows: a twin-screw setting temperature of 190 °C, a screw speed of 50 rpm, a feed speed of 30 rpm, a residence time of 90 s, a mold temperature of 30 °C, and an injection pressure of 0.60 MPa, with a holding pressure of 0.30 MPa and holding time range of 35–65 s (adjusted to the type of test specimen). Tensile, flexural, impact, and rheological specimens were prepared for further research. Abbreviations of the sample names are listed in Table 1.

### 2.3. Characterization

#### 2.3.1. Scanning Electron Microscopy (SEM) Observations

The specimens quenched in liquid nitrogen were coated with gold and cross-section parts were observed using an SEM S4800 apparatus (Rigaku Corporation, Tokyo, Japan) at an accelerating voltage of 5 kV.

#### 2.3.2. Mechanical Tests

The tensile and flexural properties PP/lignin blends were characterized using an AI-7000-NGD testing machine (Goodtechwill, China). Crosshead speeds of 10 and 1.5 mm min^−1^ were used for the tensile tests (ASTM standard D638) [19] and for the flexural tests (ASTM standard D790) [20], respectively. The notched Izod impact strength measurements (ASTM standard D256) [21] were performed using a digital Izod Impact Testing Machine FBS-5.5D (Furbs Testing Equipment Co., Ltd., Xiamen, China) equipped with a 2.75 J Izod impact pendulum. All the tests for each recipe were repeated six times and the average was considered as the characteristic value.

To compare the clarity of mechanical performance improvement comparison, specific mechanical performance tests were calculated and analyzed using the following equations:

RTS = (TS1 − TS0)/TS0 × 100%,(1)

RFS = (FS1 − FS0)/FS0 × 100%,(2)

RIS = (IS1 − IS0)/IS0 × 100%,(3)

Here, RTS, RFS, and RIS represent the relative tensile, flexural, and impact strength, respectively. TS1, FS1, and IS1 denote the tensile, flexural, and impact strength of PP/Lignin blends with CPA, while TS0, FS0, and IS0 represent the corresponding strengths of PP/lignin blends without CPA.

#### 2.3.3. Density and Melt Flow Index (MFI)

The densities (*ρ*) of all samples were measured on an electronic densimeter MH-300A (Furbs Testing Equipment Co., Ltd., Xiamen, China). The MFI of the neat PP and PP–lignin composites were determined according to ASTM D1238 [22] at 230 °C with a load of 2.16 kg using a Melt Flow indexer (FBS-400B, Furbs Testing Equipment Co., Ltd., Xiamen, China).

#### 2.3.4. Heat Deflection Temperature (HDT)

The Heat Deflection Temperature (HDT) was measured using a thermal deformation Vicat softening point temperature tester HDT/V-1104, according to ASTM D648 [23] at 0.45 MPa load, and a heating rate of 2 °C min^−1^ using the three-point bending cantilever.

#### 2.3.5. Thermal Analysis

The differential scanning calorimetry (DSC) analysis was performed in a TA Instruments DSC25 setup under N_2_ flow (flow rate: 50 mL min^−1^). Around 4–6 mg of the sample was placed in a 100 μL aluminum pan. Crystallization peak temperature (*T_c_*), melting temperature (*T_m_*), crystallization enthalpy (Δ*H_c_*), and the melting enthalpy (Δ*H_m_*) of the specimen were determined in the heat/cool/heat cycle method (heated ramp of 10 °C min^−1^ from −50 to 250 °C then cooled at the same rate). The degree of crystallinity (*X_c_*) of PP and PP–lignin composites was calculated according to the following equation:

*Xc* = Δ*H_m_*/(φΔH_m_*) × 100%, (4)

where Δ*H_m_* is the melting enthalpy (J g^−1^) calculated from the integral area of melting peak, *φ* is the weight fraction of PP in the composite, and ΔH_m_* is the theoretical melting enthalpy of 100% crystalline PP (207.1 J g^−1^) [24,25].

#### 2.3.6. Rheological Test

The rheological analysis of neat PP and PP/KL blends was conducted on a Dynamic Rheometer (Ar2000ex, TA Instruments, New Castle, DE, USA) with a gap of 1 mm between the parallel plates (25 mm). The dynamic frequency sweep test was carried out from an initial frequency of 0.1 Hz to a final frequency of 100 Hz with a strain of 1% at 190 °C to determine the complex viscosity (η*), as well as the storage (G′) and loss (G″) moduli of the samples. The dynamic temperature sweep test was performed using a fixed shear strain of 1% and an oscillation frequency of 10 rad/s, with a temperature range of 190–290 °C at a heating rate of 5 °C min^−1^. All experiments were conducted in a nitrogen atmosphere to ensure controlled environmental conditions.

## 3. Results and Discussion

### 3.1. Morphological Observations

**Figure 1:**
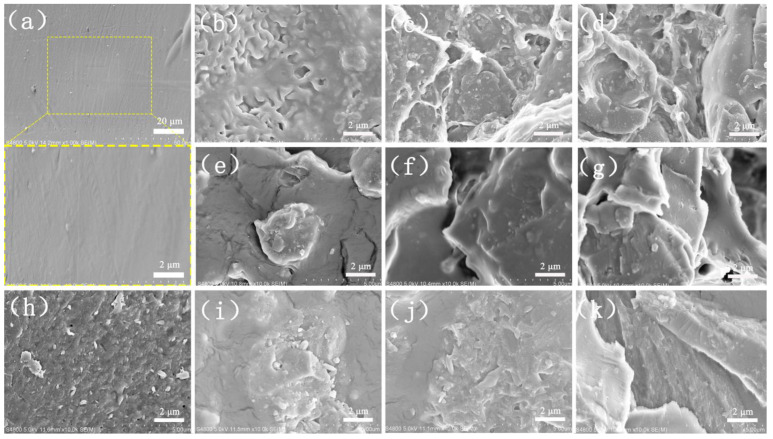
SEM images of (**a**) neat PP, (**b**) P80AL20, (**c**) P60AL40, (**d**) P40AL60, (**e**) P80KL20, (**f**) P60KL40, (**g**) P40KL60, (**h**) P60KL40-10C1, (**i**) P60KL40-10C2, (**j**) P60KL40-10C3, and (**k**) P60KL40-10C4.

The morphologies of neat PP and PP/lignin blends were first observed using SEM, as shown in Figure 1. The cross-section of neat PP exhibits a smooth and flat surface (Figure 1a). With AAL addition, rough surfaces with cracks presented on the fracture section of blends, and the edges became clear when the AAL content increased (Figure 1b–d). The presence of these cracks and edges indicated a low compatibility of AAL and the PP matrix [26,27]. The same trend was prevalent in the PP/KL blends (Figure 1e–g). When MAPP was introduced into P60KL40, the surface became smooth again and those cracks were mostly eliminated, suggesting an improved compatibility between PP and KL when using MAPP (Figure 1h–k).

### 3.2. Mechanical Properties

**Figure 2:**
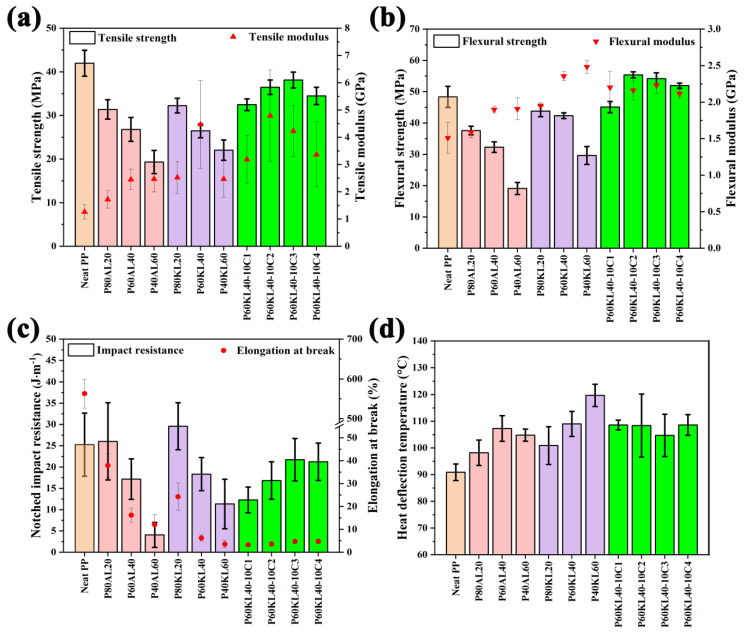
The mechanical properties of the PP/Lignin blends with/without compatibilizers: (**a**) Tensile properties; (**b**) Flexural properties; (**c**) Notch impact properties; and (**d**) HDT values.

The mechanical performances of various PP/lignin blends are shown in Figure 2 and the corresponding data are summarized in Table S3. Neat PP displayed 42.0 MPa and 1257 MPa in tensile strength and modulus and 48.3 MPa and 1508 MPa in flexural strength and modulus. With the AAL addition, the tensile and flexural moduli increased up to 2451 MPa and 1897 MPa at P40AL60. This revealed that AAL worked as a stiff filler and increased the modulus of the blends. However, their elongation at break decreased to 12.03% and the impact strength also decreased from 25 J m^−1^ to 4 J m^−1^. This may suggest poor compatibility between PP and AAL, which further induced an easy crack propagation during fracture.

The PP/KL blends displayed slightly higher tensile and flexural properties compared to corresponding PP/AAL blends. This could be attributed to two factors: (1) the particle size of KL is smaller than that of AAL; (2) the large polydispersity index value and similar Mw value of KL, where the small molecular fractions of KL contributed to better dispersity of KL in the PP matrix than AAL (Table S1). However, the lignin content is too low to achieve our aim. The performance of the blends largely decreased and displayed a very low MFI (less than 10 g per 10 min) to carry out processing at P40KL60. Therefore, we focused on P60KL40 to carry out the following experiments.

Four different MAPP samples were used to increase the compatibility of P60KL40.With 10 pph addition of CPA1 and CPA4, the mechanical PP/KL blends had a certain increase in tensile and flexural performance. Notably, the tensile strengths of P60KL40-10C2 and P60KL40-10C3 increased to 36.5 MPa and 38.1 MPa (ca. 91% of neat PP), and the flexural strengths were improved to 55.4 MPa and 54.2 MPa (ca. 155% of neat PP). The modulus increases were even higher, which were up to 336% and 143% of neat PP for tensile and flexural modulus, respectively. In addition, the optimized impact resistance was increased from 18.3 J m^−1^ at P60KL40 to the best performance of 21.7 J m^−1^ at P60KL40-10C3. These results suggest that CPA3 with high MA levels (≥0.8 wt.%) and moderate MFI (60–100 g 10 min^−1^) is favorable in PP/KL blend reinforcement.

The HDT of all specimens are shown in Figure 2d. The HDT of neat PP was 90.9 °C. With the addition of lignin, the values were both increased, to 107.3 °C and 109.0 °C for P60KL40 and P60AL40, respectively. It further reached a maximum value of 119.7 °C at P40KL60, which was due to the high thermal stability of KL. As CPAs were incorporated, a slight HDT decrease, within 4.3 °C, could be observed, indicating a loss in stiffness at elevated temperature because of the rubbery phase presented [28]. Considering that CPA2 and CPA3 provide better mechanical performance for PP/KL blends, the following analyses concentrate on these two compatibilizers.

### 3.3. Thermal Analysis

**Figure 3:**
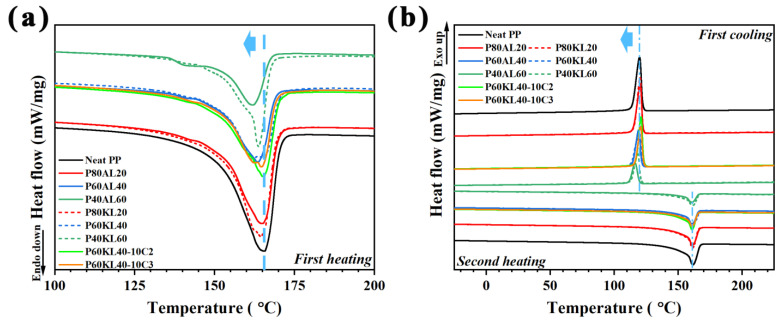
The DSC of cooling and heating cycles of neat PP and PP/lignin blends with/without compatibilizers. (**a**) First heating; (**b**) First cooling and second heating.

Differential scanning calorimetry (DSC) was performed to characterize the non-isothermal crystallization and melting behavior of various PP/lignin blends, and the data are summarized in Table 2. A melting peak at 165.51 °C for neat PP indicates the ɑ-crystalline of PP. With the increasing amounts of AAL, this melting peak shifted to a lower temperature of 161.88 °C for P40AL60, and a new boarder weak melting peak emerged around 150 °C (Figure 3), indicating the transformation of PP from the α phase to the β-crystalline phase [29]. In addition, P40AL60 exhibited lower *T_c_* and a higher undercooling degree than other specimens, which led to a higher crystallization driving force at larger addition amounts of AAL. Thus, AAL addition induced high density and growth rate of PP crystals, contributing to the observed increase in moduli in mechanical tests.

Compared with AAL, the KL additions presented similar effects on PP crystallization, where Tm decreased to 161.99 °C at P60KL40. Notably, P60KL40 showed a little higher Δ*H_m_* of 64.64 J g^−1^ and *X_c_* of 52.02% than P60AL40, suggesting KL restricted the movement of the PP segments less and maintained higher regularity of the PP chain structure. When 10 pph of CPA2 was used, both Δ*H_m_* and *X_c_* of P60KL40-10C2 increased from 64.64 J g^−1^ and 52.02% to 73.72 J g^−1^ and 65.26%, respectively. This reveals that the presence of CPA2 enhanced the interaction between the PP and KL, which further increased the crystallinity of PP. P60KL40-10C3 displayed a few lower values, where Δ*H_m_* and *X_c_* were 67.49 J g^−1^ and 59.74%, respectively. The results demonstrated that both CPAs improved compatibility between the KL and the PP matrix, and the effect varied based on the MA level and MFI values.

### 3.4. Rheological Characterization

**Figure 4:**
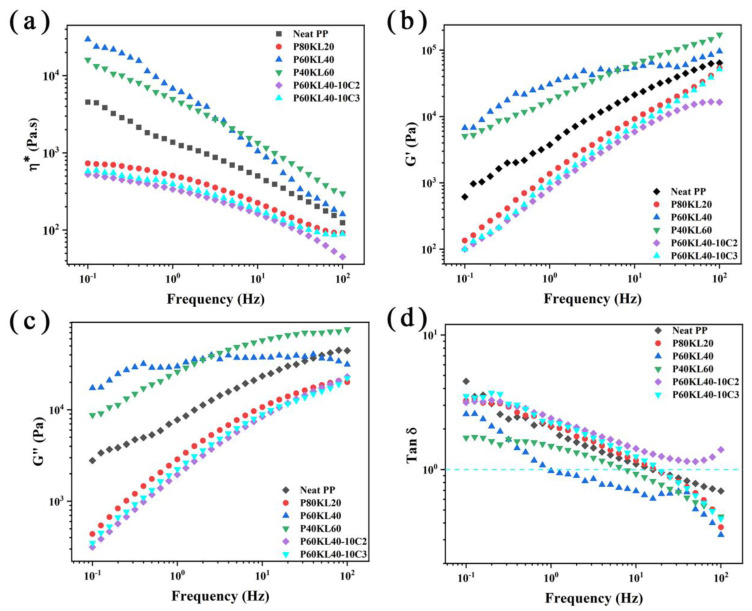
Rheological behavior of PP/KL blends with/without compatibilizers. (**a**) Complex viscosity (η*), (**b**) storage modulus (G′), (**c**) loss modulus (G″), and (**d**) tan δ at different frequencies.

To elucidate the interaction between lignin and PP, as well as the influence of compatibilizer on the PP/lignin blend system, the angular-frequency-dependent complex viscosity moduli of the specimen melts were measured at 190 °C, and the results are shown in Figure 4a. The neat PP and PP/lignin melts were all non-Newtonian fluids according to the shear-thinning behavior and Newtonian plateau at low frequency. With the increased amounts of KL, the complex viscosity of PP/KL blends first decreased for P80KL20. This value turned to an increase above 20% KL addition, as shown in Figure 4a. This can be explained by the following hypothesis: the platelet/lamellar structure that KL particles at low weight fraction can slide against each other during the application of shear forces and further increase flowability in the PP/KL blends; this is consistent with other reports [30]. In contrast, when the addition of lignin is even higher, the infusible KL particles hinder the flowability of PP/lignin blends, which induces the viscosity increase [31]. When compatibilizers were added in the blends, the complex viscosities of both P60KL40-10C2 and P60KL40-10C3 at all frequencies were lower than that of neat PP. This suggests that both MAPP compatibilizers effectively improved the miscibility of both PP and KL phases.

The storage (G′) and loss (G″) moduli of the PP/lignin composites with substantial lignin addition were roughly on the same orders of magnitude across the whole frequency range. The G′ and G″ of the P40KL60 specimens were higher than neat PP at all frequencies, as seen in Figure 4b, c. The presence of a large amount of lignin significantly impedes the relaxation process of the PP matrix. As shown in Figure 4d, the frequency of tan δ = 1 in the PP/lignin blends was lower than that of neat PP (15.66 Hz), which leads to a weak liquid-like behavior at lower frequencies [32]. Meanwhile, when compatibilizers were added to the blends, the crossover point of P60KL40-10C3 rose to 19.95 Hz and tan δ of P60KL40-10C2 above 1 across the whole frequency range. This indicates an enhanced compatibility of lignin and PP phases. Considering that CPA3 has a lower MFI value than CPA2, this suggests that the polymer chains in P60KL40-10C3 are relatively entangled, further benefiting the mechanical performance and processability improvements.

**Figure 5:**
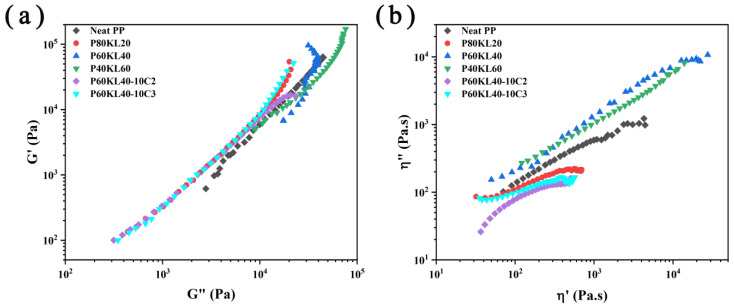
Influence of lignin on the rheological properties of PP with/without compatibilizers. (**a**) Han’s plot (G′-G″), (**b**) Cole–Cole (η′-η″).

To further investigate the compatibility of lignin and PP, the rheological data were then analyzed using the time-concentration superposition principles. Figure 5a shows Han’s plots (G′-G″), which represent the compatibility in different recipes. The slope of P80KL20 slightly departed from that of neat PP, while it changed remarkably for P60KL40 and P40KL60, demonstrating the poor compatibility between lignin particles and the PP matrix above 20% KL addition [33]. In addition, the slope of P60KL40-10C3 was essentially the same as the PP matrix and was even more pronounced in P60KL40-10C2, indicating enhanced compatibility by using CPA2 and CPA3. The curve of storage viscosity (η″, imaginary part of η*) against the dynamic viscosity (η′, real part of η*), so-called Cole–Cole curves, were further used to analyze the PP/lignin with/without compatibilizers in the multiphase systems (Figure 5b). The PP/lignin blends with compatibilizers showed low-frequency warping at the end of the curve in the high η′ region. This can be taken as a sign of the formation of a heterogeneous network structure, where the MA side of MAPP chemically bonded lignin molecules and the PP parts physically associated with PP regions [34]. Thus, the network structure resulted in a low-frequency relaxation behavior and an increase in the relaxation time [35,36].

### 3.5. Comparative Analysis of Research

**Figure 6:**
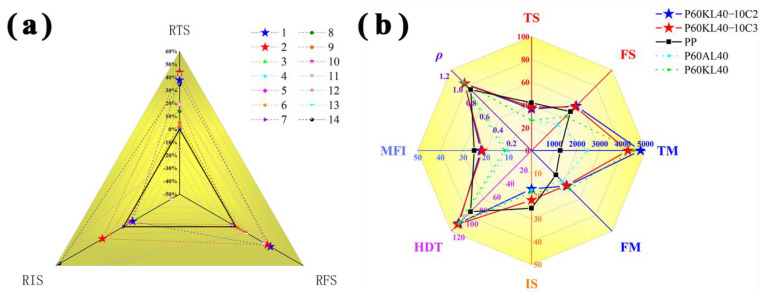
(**a**) The relative tensile, flexural, and impact strength with related studies and (**b**) comparison illustration of the fundamental performance. ★: this work.

The significant enhancement in the mechanical properties presented in this study, along with its comparison to previous reports, is illustrated in Figure 6 and summarized in Table S4. Due to the high MA levels and moderate MFI values of CPA3, the compatibility of PP and lignin achieved the optimum, and the relative tensile, flexural, and impact strengths were all increased by at least 18.6% based on the corresponding PP/KL blends (Figure 6a). The improvements achieved using this method surpass the outcomes obtained from other diverse recipes documented in published reports (Table S3). Meanwhile, P60KL40-10C3 gained large moduli, while other properties maintained similar values as neat PP (Figure 6b). The reason can be summarized as follows. Both KL and AAL displayed poor compatibility with the PP matrix. As a result, this resulted in crack propagations during deformation and significantly influenced the mechanical properties of the PP/lignin blends. When MAPP compatibilizers were incorporated, PP and lignin phases were chemically and physically bonded and interconnected into a network structure, improving the compatibility of all components and resulting in a relatively smooth surface. The MA level determines the reaction efficiency between MAPP and the lignin molecules, and MFI reflects the chain length of the PP segment, which further affects the cross-link density between PP and lignin phases. In terms of mechanical performance, CPA with high MA levels (≥0.8 wt.%) and moderate MFI (60–100 g 10 min^−1^) are favorable for constructing a reinforced network. Meanwhile, in the aspect of decreasing fossil feedstock use and related GHG emissions, PP/KL blends provide an approximately 40% reduction in PP landfill even without recycling, which is comparable to the current technique for PP recycling.

Considering the cost discrepancies among PP (USD 1168–1515 per MT), CPA3 (USD 2700–3500 per MT), and lignin (USD 100–300 per MT), a 20% reduction in expenses can be achieved by utilizing PP/lignin blends. Therefore, cost-effective high-lignin-filled PP not only has the potential to replace its corresponding fossil-based counterpart, but also contributes to a reduction in the amount of PP sent to landfills. This dual benefit of cost savings and waste reduction emphasizes the promising prospects for the substantial utilization of technical lignin in industry, thereby adding significant value to this renewable resource.

## 4. Conclusions

In this study, we explored the blending of up to 60 wt.% lignins with PP using twin-screw extrusion and injection molding techniques. The introduction of lignin resulted in increased tensile and flexural moduli, albeit with a rapid decrease in impact strength. Additionally, PP/KL blends displayed slightly superior tensile and flexural properties compared to PP/AAL blends due to the better dispersity of KL. When MAPP compatibilizers were incorporated, PP and KL phases were chemically and physically bonded, forming an interconnected network structure. Specifically, CPA with high MA levels (≥0.8 wt.%) and moderate MFI (60–100 g 10 min^−1^) proved favorable for constructing a reinforced network. As a result, P60KL40-10C3 demonstrated optimal mechanical properties, such as tensile strength of 38.1 MPa, tensile modulus of 4227 MPa, flexural strength of 54.2 MPa, flexural modulus of 2160 MPa, and impact strength of 21.7 J m^−1^, which were comparable to previous studies and neat PP. Considering the high filling content of lignin, utilizing these blends can lead to a 20% reduction in cost. Therefore, this study not only provides a relatively sustainable and cost-effective way of replacing commercial PP plastics, but also holds promising prospects for value addition of lignin.

## Figures and Tables

**Table 1:** The sample names and characteristics of different composite blends.

Sample Names	PP (wt.%)	AAL (wt.%)	KL (wt.%)	CPA1 (pph) ^a^	CPA2 (pph) ^a^	CPA3 (pph) ^a^	CPA4 (pph) ^a^
neat PP	100						
P80AL20	80	20					
P60AL40	60	40					
P40AL60	40	60					
P80KL20	80		20				
P60KL40	60		40				
P40KL60	40		60				
P60KL40-10C1	60		40	10			
P60KL40-10C2	60		40		10		
P60KL40-10C3	60		40			10	
P60KL40-10C4	60		40				10

^a^ pph is parts per hundreds of PP/lignin blends.

**Table 2:** The DSC parameters of the heating and cooling processes of neat PP and PP–lignin composites with/without compatibilizers.

Sample	1st Heat			2nd Heat			1st Cool	
	*T_m_* (°C)	Δ*H_m_* (J g^−1^)	*X_c_* (%)	*T_m_* (°C)	Δ*H_m_* (J g^−1^)	*X_c_* (%)	*T_c_* (°C)	Δ*H_c_* (J g^−1^)
neat PP	165.51	99.79	48.18	161.49	121.5	58.67	119.79	118.80
P80AL20	164.78	84.21	50.83	161.15	99.11	59.82	120.30	97.07
P60AL40	163.44	59.92	48.22	160.56	69.39	55.84	119.05	68.04
P40AL60	161.88	42.04	50.75	160.77	40.42	48.79	116.32	39.25
P80KL20	164.43	91.07	54.97	161.17	106.80	64.46	120.20	105.00
P60KL40	161.99	64.64	52.02	160.86	75.20	60.52	119.51	76.91
P40KL60	163.74	55.44	66.92	161.32	57.91	69.91	118.21	58.54
P60KL40-10C2	165.04	73.72	65.26	160.83	83.18	73.63	120.96	82.21
P60KL40-10C3	164.60	67.49	59.74	160.76	70.34	62.27	120.62	71.16

## Data Availability

Data are contained within the article or supplementary material.

## Supplementary Materials

The following supporting information can be downloaded at: https://www.mdpi.com/article/10.3390/polym16040520/s1, Figure S1: The dynamic temperature sweep of PP/lignin blends with/without compatibilizers. (a) Complex viscosity (η*), (b) storage modulus (G′), (c) loss modulus (G″); Figure S2: KL (a) and AAL (b) particle size distribution; Table S1: Relative molecular weight and functional group content of lignin; Table S2: The maleic anhydride (MA)-functionalized polypropylene copolymer product datasheet; Table S3: Mechanical properties and heat deflection temperature (HDT) of PP/AAL or KL blends with/without compatibilizers; Table S4: Comparison of the tensile, flexural, and impact properties of the lignin-based composites with compatibilizers. References [37,38,39,40,41,42,43,44,45] are cited in the supplementary materials.
