# Supplementary material for: Enhancing Mechanical Performance of High-Lignin-Filled Polypropylene via Reactive Extrusion
Source: Polymers (Basel). 2024 Feb 14;16(4):520. doi: 10.3390/polym16040520 (PMC11154581; doi:10.3390/polym16040520)
Supplement: Supplementary file 1 [file polymers-16-00520-s001.zip › polymers-2833509-supplementary.pdf]

Supplementary Materials

Enhancing Mechanical Performance of High-Lignin-Filled Polypropylene via Reactive Extrusion

Ruichen Wang, Xiangyu You *, Shijie Qi, Ruiyun Tian and Huijie Zhang *

College of Bioresources Chemical and Materials Engineering, Shaanxi University of Science of Science & Technology, Xi'an 710021, China; wang.ruichen@sust.edu.cn (R.W.); 210111015@sust.edu.cn (S.Q.); 230111094@sust.edu.cn (R.T.)

* Correspondence: xyyou@sust.edu.cn (X.Y.); hjzhang@sust.edu.cn (H.Z.)

CONTENTS:

The analysis of the dynamic temperature sweep S-2

Supporting Tables and Figures S-4

Reference S-8

1. The analysis of the dynamic temperature sweep

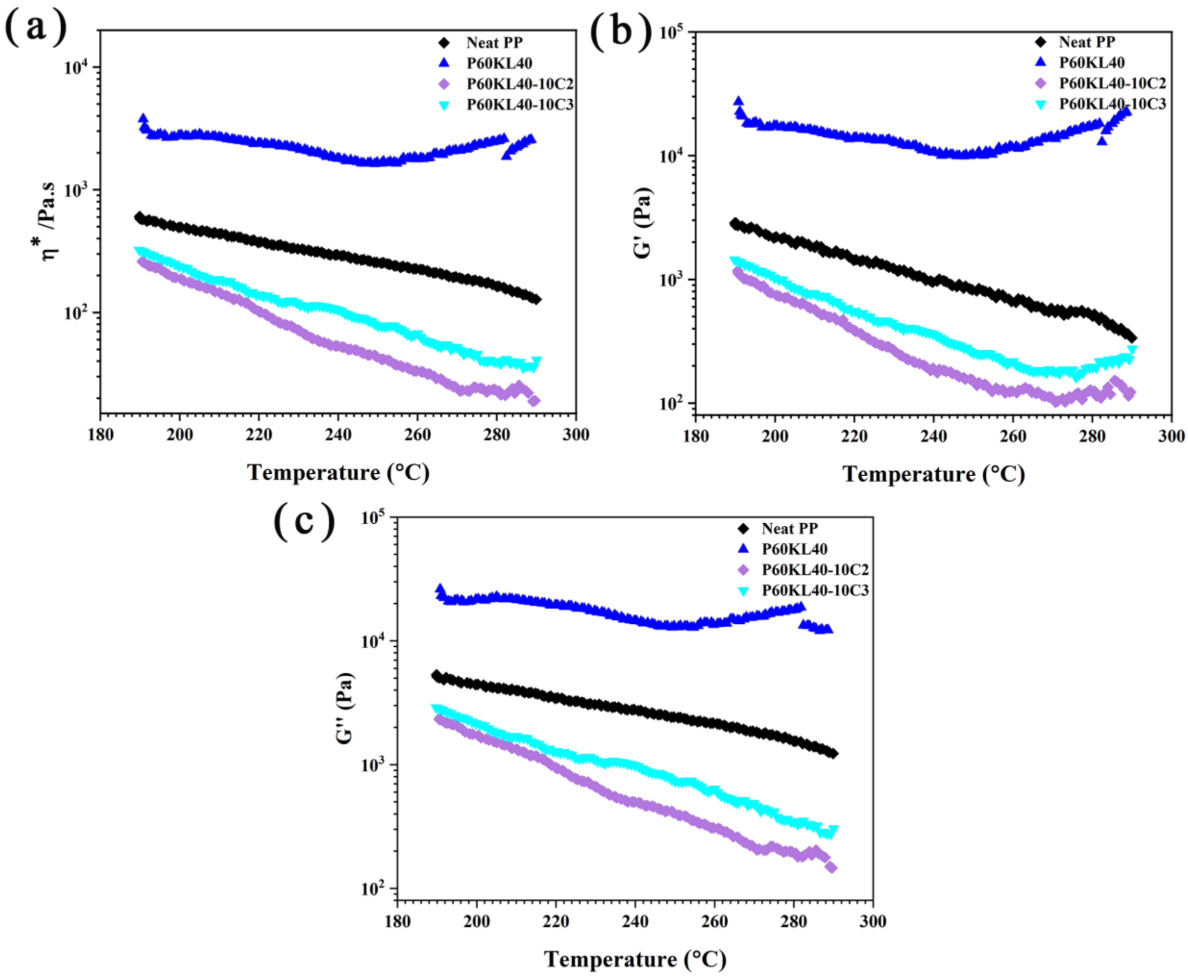

Figure S1. The dynamic temperature sweep of PP/Lignin blends with/without campatibilizers. (a) complex viscosity (η*), (b) storage modulus (G'), (c) loss modulus(G").

The discrepancy of the PP/lignin blends with/without compatibilizers is further analyzed through the dynamic temperature sweep test, as shown in Figure S1. The complex viscosity and storage modulus of the PP/lignin blends were larger and less variable than the PP matrix, which had a monotonically decreasing tendency across the temperature sweep range. However, the complex viscosity and storage modulus of the PP/lignin composites with compatibilizers decline faster with increasing temperature because the melt of the PP/lignin/compatibilizers blends was more likely to persist in the viscous liquid state. It is advantageous to restrain the entanglement of long chains and improve fluidity via lignin's coupling to the PP molecular chain.[37]

2. Supporting Tables

Table S1. Relative molecular weight and functional groups content of lignin

**Sample**				**mmol g^{-1} of lignin**		
	**M_n (g mol^{-1})**	**M_w (g mol^{-1})**	**PDI**	**Ar-OH**	**Alk-OH**	**-OH**
**KL**	975	6305	6.5	4.3	0.0	4.3
**AL**	3700	5900	1.6	0.0	0.2	0.2

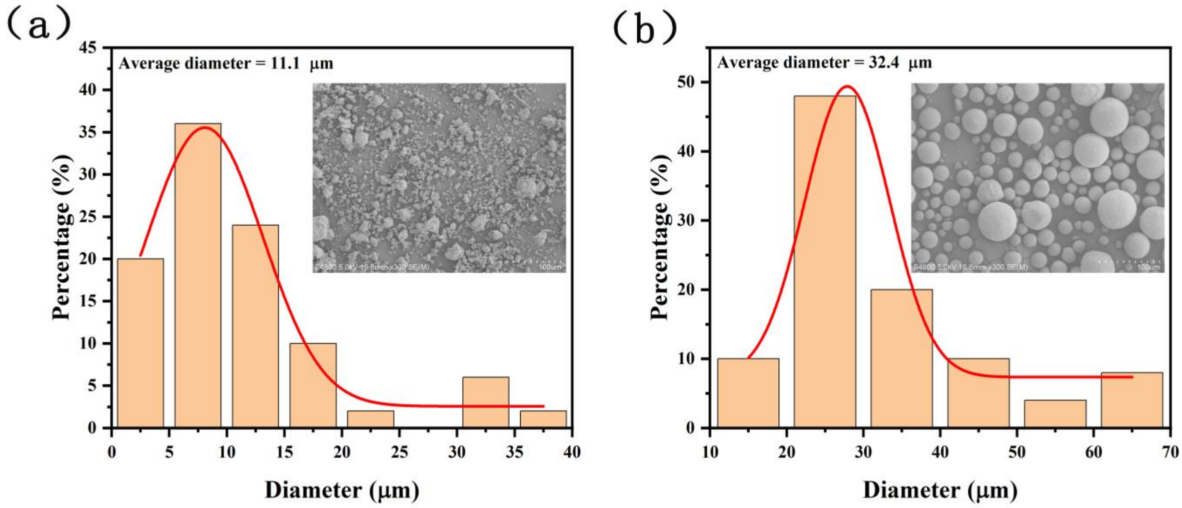

Figure S2. KL (a) and AAL (b) particle size distribution.

Table S2. The maleic anhydride (MA) functionalized polypropylene copolymer product datasheet

Abbr.[1]	The product grade	Density (g/cm^3)	MA level	Maleic anhydride grafted monomer average number of molecules [N_m](calculated)*	Melt Mass-Flow Index(MFI) (190°C/2.16kg) ASTM 1238
CPA1	ExxonMobil EXXELOR™ PO 1015	0.90	0.25~ 0.50wt.%	664~1331	22 g/ 10min
CPA2	ExxonMobil EXXELOR™ PO 1020	0.90	0.50~ 1.0wt.%	331~664	112 g/ 10min
CPA3	Coace® B1	0.92	≥ 0.8wt.%	≤ 414	60-100 g/ 10min
CPA4	Coace® B1R	0.92	≥ 0.8wt.%	≤ 414	1-4 g/ 10min

[1]It is a standardised abbreviation used for words in scientific citations.

The datasheet obtained from the Official website of ExxonMobil and COACE CHEMICAL CO.,LTD.

* The calculated equation is N_m = [(98/MA level-98)/42]

98 and 42 are the relative molecular mass of maleic anhydride and polypropylene monomer, respectively

Table S3. Mechanical properties and heat deflection temperature(HDT) of PP/AAL or KL Blends with/without Compatibilizers

Sample	**Tensile strength (σ_m, MPa)**	**Tensile Modulus (E_t, MPa)**	**Elongation at yield (ε_y, %)**	**Elongation at break (ε_b, %)**	**Flexural Strength (σ_f, MPa)**	**Flexural Modulus (E_f, MPa)**	**Noched Impact Resistance ($J{\cdot}m^{-1}$)**	**HDT (℃)**
neat PP	42.0 (±2.98)	1257 (±259.8)	10.12 (±2.16)	562.96 (±36.44)	48.3 (±3.36)	1508 (±210.3)	25.3 (±7.41)	90.9 (±3.11)
P80AL20	31.4 (±2.22)	1715 (±317.3)	4.80 (±0.86)	37.89 (±5.18)	37.6 (±1.35)	1588 (±78.4)	26.0 (±9.05)	98.2 (±4.74)
P60AL40	26.8 (±2.72)	2451 (±376.9)	3.57 (±0.62)	16.19 (±3.04)	32.3 (±1.69)	1897 (±47.2)	17.2 (±4.76)	107.3 (±4.81)
P40AL60	19.3 (±2.65)	2466 (±476.1)	3.93 (±0.93)	12.03 (±4.33)	19.1 (±1.93)	1908 (±151.9)	4.1 (±2.92)	104.8 (±2.26)
P80KL20	32.3 (±1.68)	2527 (±577.7)	4.32 (±0.43)	24.24 (±5.96)	43.8 (±1.75)	1952 (±38.5)	29.6 (±5.51)	100.9 (±7.07)
P60KL40	26.5 (±1.57)	4472 (±1607.5)	2.97 (±0.71)	6.19 (±1.33)	42.4 (±0.91)	2356 (±60.5)	18.3 (±3.89)	109 (±4.67)
P60KL40-10C1	32.5 (±1.34)	3187 (±879.2)	3.08 (±0.14)	3.26 (±0.23)	45.1 (±1.80)	2483 (±89.7)	12.3 (±3.03)	108.6 (±1.84)
P60KL40-10C2	36.5 (±1.66)	4787 (±1673.1)	3.39 (±0.39)	3.57 (±0.55)	55.4 (±0.98)	2201 (±214.7)	16.8 (±4.40)	108.4 (±11.81)
P60KL40-10C3	38.1 (±1.83)	4227 (±938.6)	3.75 (±0.12)	4.70 (±0.48)	54.2 (±1.85)	2160 (±131.5)	21.7 (±4.97)	104.7 (±7.92)
P60KL40-10C4	34.5 (±1.98)	3365 (±1192.5)	4.16 (±0.40)	4.70 (±0.68)	51.9 (±0.82)	2231 (±117.4)	21.3 (±4.38)	108.6 (±3.89)
P40KL60	22.0 (±2.33)	2466 (±663.4)	2.64 (±0.60)	3.52 (±1.56)	29.6 (±2.84)	2483 (±89.7)	11.3 (±5.81)	119.7 (±4.17)

Table S4. Comparison of the Tensile, Flexural and Impact Properties of the lignin-based composites with Compatibilizers

Number	Lignin content (wt.%)	Compatibilizers (CPA)		Relative Tensile Strength (RTS)	Relative Flexural Strength (RFS)	Relative Impact Strength (RIS)	Refere-nce
		Type	Content (phc)[a]				
1	40	CPA2	10	+37.74%	+30.66%	-8.20%	This work
2	40	CPA3	10	+43.77%	+27.83%	+18.57%	
3	2	PB	9.8	-1.60%	/	/	[38]
4	2	lignin-FD@50	2	-0.52%	/	/	[39]
5	3	ZnO	0.6	+1.76%	/	/	[40]
6	10	PP-g-MA	3	+1.83%	+4.81%	+12.02%	[41]
7	15	CE	15	-0.02%	/	-0.05%	[42]
8	20	MAPP (ME42035)	2.5	+13.78%	/	/	[43]
9	30	MAPE	5	+5.00%	+1.43%	-5.26%	[44]
10	30	MAPP	3	+18.79%	-3.09%	-4.95%	[17]
11	30	MAPP	5	+20.00%	+25.00%	-42.86%	[18]
12	30	EBGMA	5	-15.56%	+9.38%	+85.71%	
13	30	PEGMA	17.7	+42.85%	/	/	[45]
14	30	BCFS	2	+34.34%	/	+56.76%	[26]

a) The meaning of ‘phc’ is parts per hundreds of Lignin/Polymer composites.
The abbreviations of the CPA in the table are sourced from relevant reference.

References

17. Dias, O.A.T.; Sain, M.; Cesarino, I.; Leão, A.L. Development of high bio-content polypropylene composites with different industrial lignins. *Polym. Adv. Technol.* **2018**, *30*, 70–78, https://doi.org/10.1002/pat.4444.
18. Abdelwahab, M.A.; Misra, M.; Mohanty, A.K. Injection molded biocomposites from polypropylene and lignin: Effect of compatibilizers on interfacial adhesion and performance. *Ind. Crop. Prod.* **2019**, *132*, 497–510, https://doi.org/10.1016/j.indcrop.2019.02.026.
26. Xu, X.; He, Z.; Lu, S.; Guo, D.; Yu, J. Enhanced thermal and mechanical properties of lignin/polypropylene wood-plastic composite by using flexible segment-containing reactive compatibilizer. *Macromol. Res.* **2014**, *22*, 1084–1089, https://doi.org/10.1007/s13233-014-2161-3.
37. Liu, Y.; Tian, B.; Liu, X. Preparation of polypropylene blends with the long chain branched behavior through reactive blending induced by pre-irradiation. *Radiat. Phys. Chem.* **2020**, *177*, 109188, https://doi.org/10.1016/j.radphyschem.2020.109188.
38. Chen, F.; Zhang, M. Maleic anhydride-modified polyolefins as compatibilizer for lignin-reinforced polypropylene composites. *Polym. Compos.* **2018**, *40*, 2594–2601, https://doi.org/10.1002/pc.25053.
39. Chen, F.; Liu, W.; Shahabadi, S.I.S.; Xu, J.; Lu, X. Sheet-Like Lignin Particles as Multifunctional Fillers in Polypropylene. *ACS Sustain. Chem. Eng.* **2016**, *4*, 4997–5004, https://doi.org/10.1021/acssuschemeng.6b01369.
40. Klapiszewski, .; Grząbka-Zasadzińska, A.; Borysiak, S.; Jesionowski, T. Preparation and characterization of polypropylene composites reinforced by functional ZnO/lignin hybrid materials. *Polym. Test.* **2019**, *79*, https://doi.org/10.1016/j.polymertesting.2019.106058.
41. Dias, O.; Negrão, D.; Silva, R.; Funari, C.; Cesarino, I.; Leao, A. Studies of lignin as reinforcement for plastics composites. *Mol. Cryst. Liq. Cryst.* **2016**, *628*, 72–78, https://doi.org/10.1080/15421406.2015.1137677.
42. Maldhure, A.V.; Ekhe, J.D.; Deenadayalan, E. Mechanical properties of polypropylene blended with esterified and alkylated lignin. *J. Appl. Polym. Sci.* **2012**, *125*, 1701–1712, https://doi.org/10.1002/app.35633.
43. Abdelwahab, M.; Misra, M.; Mohanty, A. Effect of maleated polypropylene emulsion on the mechanical and thermal properties of lignin-polypropylene blends. PROCEEDINGS OF PPS-30: The 30th International Conference of the Polymer Processing Society – Conference Papers. LOCATION OF CONFERENCE, United StatesDATE OF CONFERENCE; p. 150006.
44. Sameni, J.; Jaffer, S.A.; Sain, M. Thermal and mechanical properties of soda lignin/HDPE blends. *Compos. Part A: Appl. Sci. Manuf.* **2018**, *115*, 104–111, https://doi.org/10.1016/j.compositesa.2018.09.016.
45. Hong, S.-H.; Hwang, S.-H. Construction and foamability of lignin-reinforced low-density polyethylene biocomposites. *Mater. Today Commun.* **2021**, *28*, 102696, https://doi.org/10.1016/j.mtcomm.2021.102696.
